# Items

**Published:** 1882-10

**Authors:** 


### Items.

---

Meteorological Data.—For the month of Aug., 1882. H. Hall, Observer, U. S. Signal Station, Atlanta, Ga.

| | | |
|---|---|---|
| Barometer | —Mean | 30.038 |
| | Highest | 30.278 on 21st. |
| | Lowest | 29.837 on 28th. |
| | Range | 0.450 |
| Thermometer | —Mean | 74.8 |
| | Highest | 90.0 on 16th. |
| | Lowest | 64.5 on 31st. |
| | Range | 25.5 |
| | Greatest Daily Range | 19.4 on 15th. |
| | Least Daily Range | 3.0 on 30th. |
| Humidity | —Mean | 79.4 |
| Dew-point | —Mean | 67.3 |
| Rainfall | —Total | 5.86 inches. |
| | Greatest Daily | 1.05 on 4th. |
| | Total Number of Days | 20 |
| Wind | —Prevailing Direction | E. |
| | Maximum Velocity..(3 days) | S W. & E. 20 miles. |

---

Among the widespread and steadily increasing race of bores, says the *Canada Med. and Surg. Journal*, a high place must be claimed for those fussy and objectionable people who, under cover of social relations, persist in endeavoring to obtain a medical opinion without payment of a fee. An old lady the other day asked an eminent London surgeon, who was seated beside her at a dinner table, what was the best "cure for corns." To this the surgeon replied: "You can adopt no better plan, my dear madam, than to grease the corn over night with a tallow candle, when I venture to say you will find the corn kicking about the bottom of your bed next morning." The old lady was profuse in her thanks, but the surgeon cut them short by adding, "I should say, my dear madam, that it would still be on your foot."

---

The *Medical Press and Circular*, London, think no country where diploma traffic is openly carried on, as it is in America, has any reason to expect recognition of its medical diplomas in Europe. Our valued trans-Atlantic cotemporary is just about right on this question. Undoubtedly many shameful farces are enacted, and many outrageous frauds are perpetrated in the name of law by the bestowment of medical diplomas upon unqualified persons. The colleges themselves cannot deny the fact, and many do not even attempt to excuse it. Some diplomas ought not to be recognized anywhere.

---

THE publication of the weekly *Bulletin* of the National Board of Health has been suspended, and the appropriation heretofore disbursed by the Board in aid of local health authorities has been diverted to and placed under the control of another medical bureau.

---

DR. G. E. SUSSDORF, formerly of Macon, contributes an interesting article to the *New York Medical Journal and Obstetrical Review*, on the parturient peculiarities of the elephant.

---

THE *Indiana Medical Journal*, published at Indianapolis, and edited by Frank C. Ferguson, M.D., is the latest addition to a very large family.

---

A COMBINATION of ergot, belladonna and iodide of iron is strongly recommended in juvenile-nocturnal urinary-incontinence.

---

DR. CHARLES SMART has been appointed Secretary of the National Board of Health, *vice* Dr. T. J. Turner, resigned.

---

THE expenditures of the National Board of Health of Germany amounted, last year, to nearly four millions of dollars.

---

WHAT does the *Pacific Medical and Surgical Journal* mean by "society doctors"?

---

JABORANDI is said to be an active galactogogue.

